# Fast fourier transform based spectral features for machine learning prediction of gamma passing rates in virtual VMAT PSQA: A feasibility study

**DOI:** 10.1002/acm2.70772

**Published:** 2026-08-27

**Authors:** Bing Yan, Hu Peng, Xudong Xue, Chichao Zheng, Aidong Wu

**Affiliations:** ^1^ School of Instrument Science and Optoelectronics Engineering Hefei University of Technology Hefei Anhui China; ^2^ Department of Radiation Oncology, The First Affiliated Hospital of USTC, Division of Life Sciences and Medicine University of Science and Technology of China Hefei Anhui China; ^3^ Department of Radiation Oncology, Hubei Cancer Hospital, TongJi Medical College Huazhong University of Science and Technology Wuhan Hubei China

**Keywords:** fast fourier transform, gamma passing rate, machine learning, patient‐specific quality assurance, volumetric modulated arc therapy

## Abstract

**Purpose:**

This study aimed to evaluate the feasibility of fast Fourier transform (FFT) based spectral features for predicting volumetric modulated arc therapy (VMAT) gamma passing rates (GPRs) in virtual patient‐specific quality assurance (PSQA).

**Methods:**

A total of 481 VMAT treatment plans were retrospectively collected. Multileaf collimator (MLC) trajectories and monitor units (MU) were extracted from control points (CPs) and interpolated onto a uniform gantry‐angle grid. FFT‐based power spectra were then computed to extract global, spatial‐axis, and angular‐axis spectral features characterizing modulation complexity. These spectral features, alongside 25 conventional plan complexity metrics, were used to train Random Forest (RF) and XGBoost models for GPR regression and PSQA pass/fail classification. We employed a nested cross‐validation strategy and used SHapley Additive exPlanations (SHAP) for model interpretation.

**Results:**

Spectral features and conventional plan complexity metrics correlated significantly with GPRs, particularly spatial‐axis high‐frequency energy ratios and total spectral energy. In the regression task, the XGBoost model utilizing the combined feature set achieved the lowest mean absolute error (MAE) of 1.095 ± 0.182. For classification, the RF model with combined features yielded an AUC of 0.886 ± 0.043, an accuracy of 0.844 ± 0.066, and an F1 score of 0.602 ± 0.094. SHAP analysis confirmed that spectral energy, spatial‐axis low‐ and high‐frequency energy ratios, were major contributors to predicting PSQA failures.

**Conclusions:**

FFT‐based spectral analysis provides physically interpretable features for characterizing VMAT modulation complexity. These features showed feasible performance for GPR prediction and PSQA classification. These findings support their potential utility as interpretable inputs for virtual VMAT PSQA.

## 1 INTRODUCTION

Volumetric modulated arc therapy (VMAT) is widely utilized in contemporary radiotherapy as it enables highly conformal dose delivery while significantly reducing treatment time.^1^, ^2^ However, achieving this high degree of modulation requires intricate coordination across numerous control points (CPs). This complexity increases the delivery system's susceptibility to multileaf collimator (MLC) positioning errors and mechanical uncertainties.^3^ Consequently, measurement‐based patient‐specific quality assurance (PSQA) remains mandatory prior to treatment. Following the recommendations of AAPM Task Group 218 (TG‐218), PSQA is typically evaluated using gamma analysis (3%/2 mm, 10% dose threshold), with gamma passing rate (GPR) tolerance and action limits of 95% and 90%, respectively.^4^ Despite its reliability, physical PSQA is labor‐ and resource‐intensive. These constraints become particularly prohibitive in modern adaptive radiotherapy (ART) workflows, where rapid plan verification is essential. To alleviate this clinical burden, machine learning (ML)‐based “virtual PSQA” approaches have gained significant traction for predicting GPRs prior to delivery.^5^, ^6^, ^7^, ^8^, ^9^, ^10^, ^11^

The predictive power of virtual PSQA models heavily depends on the extracted input features, which currently fall into two main categories: aperture‐based plan complexity metrics and fluence map‐based radiomics. Aperture‐based metrics, such as aperture area variability (AAV),^12^ leaf sequence variability (LSV),^12^ and edge area metric (EAM)^13^ are aggregated from geometric descriptors at individual CPs to form plan‐level summaries. Consequently, they primarily reflect macroscopic properties, providing only limited characterization of CP ordering and the temporal dynamics of MLC motion. Fluence map‐based features capture 2D dose distributions but suffer from fluence map degeneracy, which refers to the phenomenon that distinct MLC delivery patterns can produce identical or highly similar 2D fluence maps.^14^ Because both plan complexity metrics and integrated fluence maps fail to explicitly preserve the underlying continuous delivery sequence, their ability to fully characterize VMAT dynamic complexity remains inherently limited.

To overcome these limitations, we propose viewing VMAT delivery from a signal‐processing perspective. During dynamic arc delivery, MLC leaf positions and monitor units (MU) vary continuously and can be modeled as angular‐domain signals sampled as a function of gantry angle.^15^ Previous studies have shown that highly modulated plans are often associated with complex MLC motion, characterized by rapid variations in leaf speed, frequent directional changes, and severe aperture variabilities.^16^, ^17^ Such highly dynamic MLC motion and dose rate may be interpreted as containing stronger high‐frequency components. In this context, applying the fast Fourier transform (FFT) to MLC and monitor unit (MU) signals allows these complex modulation patterns to be decomposed into distinct frequency spectra. This paradigm enables a quantitative, frequency‐domain characterization of the relative contributions of low‐ and high‐frequency components extracting complementary, motion‐related information that remains uncaptured by conventional feature sets.

This study explored FFT‐based spectral feature extraction from MLC trajectories and MU signals, applying machine learning methods to predict GPRs and classify PSQA outcomes. The primary objective is to evaluate the predictive utility of these spectral features and determine their potential value as novel inputs for virtual PSQA frameworks. To the best of our knowledge, this is the first study to employ FFT‐based spectral analysis of MLC trajectories and MU signals for GPR prediction in PSQA.

## 2 MATERIALS AND METHODS

### 2.1 Data collection

A total of 481 VMAT treatment plans were retrospectively collected across various treatment sites, including brain (40 plans, 8.3%), head and neck (43 plans, 8.9%), thorax (276 plans, 57.4%), abdomen (87 plans, 18.1%), pelvis (32 plans, 6.7%), and other sites (3 plans, 0.6%). All treatment plans were generated using either Monaco or Pinnacle treatment planning systems (TPS) and delivered on an Elekta Axesse linear accelerator (Elekta AB, Stockholm, Sweden) equipped with an Agility 160‐leaf MLC (5 mm isocentric leaf width). The optimization and dose‐calculation settings for all VMAT plans were determined according to our institutional clinical protocol and adjusted based on plan complexity. Specifically, for Monaco‐generated plans, two to four arcs were used for each plan, the gantry angle increment (Inc) was set between 15 and 20, the maximum number of CPs was limited to 300 per arc, the minimum segment width ranged from 0.5 to 0.7 cm, and low or medium fluence smoothing was applied. Dose calculation was performed using the Monte Carlo algorithm with a 3 mm dose grid and a statistical uncertainty of 1% per plan. For Pinnacle‐generated plans, two to four arcs were also used for each plan. Dose calculation was performed using the adaptive convolution algorithm. The dose grid was set to 4 mm for conventional VMAT plans and 3 mm for SBRT plans. Pretreatment PSQA was performed using the ArcCHECK system (Sun Nuclear, Melbourne, FL, USA). To obtain the TPS‐calculated dose, all treatment plans were transferred to the ArcCHECK phantom and recalculated with a dose grid resolution of 3 mm in each dimension. Prior to each measurement, a 10 cm × 10 cm reference field was used for dose calibration to correct the detector response. Gamma analysis was performed using global 3%/2 mm and 2%/1 mm criteria with a 10% dose threshold, and the GPR was used as the evaluation metric.^4^ The overall dataset characteristics, including treatment‐site distribution, TPS distribution, arc numbers, CPs and GPR distributions, were summarized in Table S1.

### 2.2 Data preprocessing and trajectory reconstruction

**FIGURE 1:**
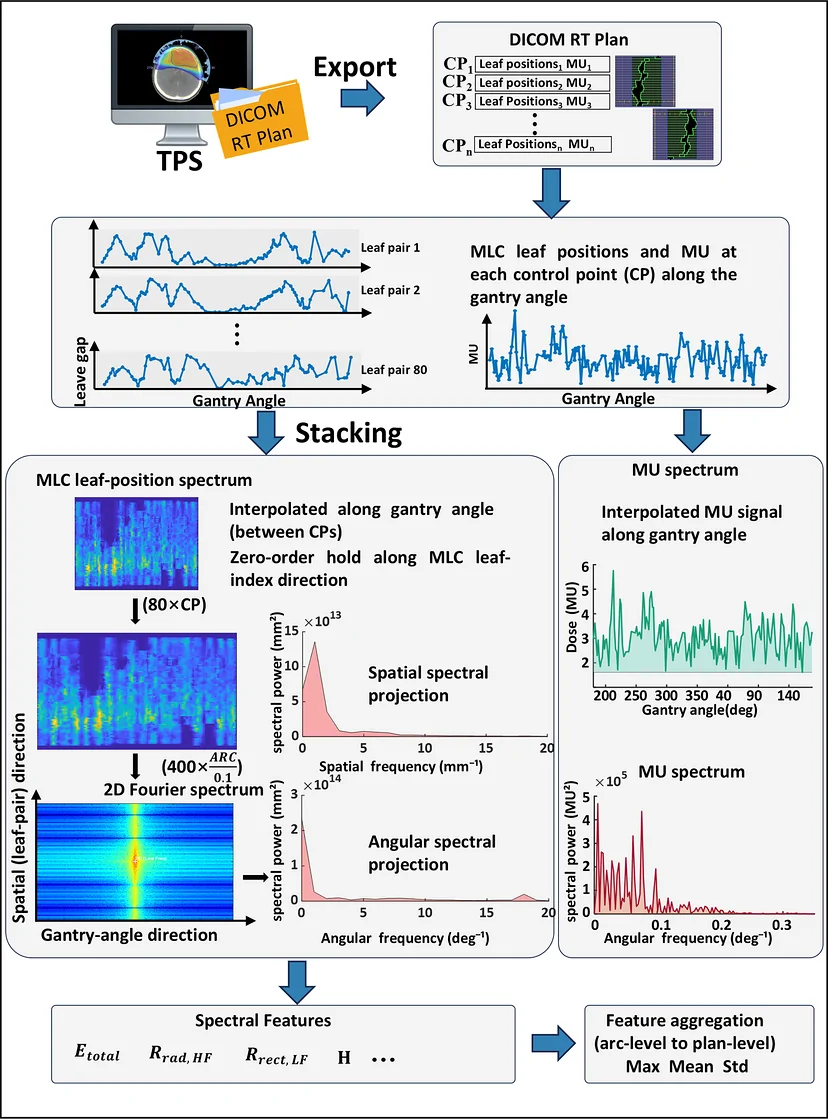
Workflow of spectral feature extraction from VMAT RT Plan data. MLC leaf positions and monitor units (MU) were extracted from CPs in the DICOM RT Plan. Leaf‐position trajectories were interpolated along gantry angle and stacked across leaf pairs to form two‐dimensional matrices, which were then transformed into 2D Fourier spectra and one‐dimensional spectral projections. MU signals were processed separately to obtain MU spectra. Spectral features were extracted at the arc level and subsequently aggregated into plan‐level features.

The overall workflow of spectral feature extraction is illustrated in Figure 1. Although VMAT delivery is inherently continuous, DICOM RT Plan files discretize this process into a sequence of CPs. In our dataset, Pinnacle‐generated plans utilized uniform gantry angle spacing (3° or 4°), whereas Monaco‐generated plans employed variable spacing (minimum 1.5°). Direct use of the CP data for spectral analysis is suboptimal for two reasons. First, because gantry speed and dose rate vary dynamically during delivery, the CP data in the DICOM RT Plan are not uniformly sampled in time. This nonuniform temporal sampling precludes direct time‐domain spectral analysis. Therefore, the gantry angle was used as a natural and consistent physical reference for VMAT delivery, allowing the MLC and MU trajectories to be reconstructed and analyzed in the angular domain. Second, the CPs in Monaco‐generated plans are not necessarily distributed at uniform gantry angle intervals, and the corresponding gantry angle positions are frequently non‐integer values. Direct use of the original CP sequence data would therefore result in a nonuniformly sampled angular‐domain signal, which is not suitable for FFT‐based spectral analysis.

To obtain a uniformly sampled angular‐domain representation for frequency‐domain analysis, we reconstructed high‐resolution MLC and MU trajectories on a uniform gantry‐angle grid. Notably, Monaco plans frequently utilized single‐beam multiple‐arc deliveries involving gantry rotation reversals.^18^ To avoid artificial discontinuities at these reversal points, CP sequences were first analyzed based on gantry‐angle monotonicity, segmenting multi‐arc beams into distinct, unidirectional arcs. Following segmentation, linear interpolation was applied to resample the MLC positions and MU data onto a uniform grid with a fine angular resolution of 0.1°.^19^, ^20^

### 2.3 2D spectral transformation

The resampled trajectories of all leaves were stacked along the spatial dimension according to their physical arrangement, forming a 2D matrix with the leaf index as the spatial axis (y) and the gantry angle as the angular axis (θ). This construction was performed independently for the left leaf bank (L), right leaf bank (R), and leaf gap (G).

Using the left leaf bank matrix f(θ,y) as a representative formulation, θ denotes the gantry angle and y denotes the spatial coordinate along the direction perpendicular to leaf motion. The data were first mean‐centered to prevent the zero‐frequency (direct‐current, DC) component from dominating the spectrum in Equation 1 as follows:

(1) $$f_{c}\left(\theta ,y\right)=f\left(\theta ,y\right)-\frac{1}{N_{\theta }N_{y}}\sum_{\theta =1}^{N_{\theta }}\sum_{y=1}^{N_{y}}f\left(\theta ,y\right)$$

where $N_{\theta }$ and $N_{y}$ denote the number of sampling points along the angular axis and the spatial axis.

Subsequently, a 2D FFT was applied, and the zero‐frequency component was shifted to the spectral center (Equation 2):

(2) $$F\left(u,v\right)=\sum_{\theta =1}^{N_{\theta }}\sum_{y=1}^{N_{y}}f_{c}\left(\theta ,y\right)\exp\left(-j2\pi \left(\frac{u\theta }{N_{\theta }}+\frac{vy}{N_{y}}\right)\right)$$

The 2D power spectrum was then defined as the squared magnitude of the Fourier transform (Equation 3):

(3) $$P\left(u,v\right)={\left|F\left(u,v\right)\right|}^{2}$$

### 2.4 Spectral feature engineering

Based on the computed power spectra, we extracted three hierarchical sets of frequency‐domain features to quantify VMAT modulation complexity:

1. Global Statistical Features

To characterize the overall spectral shape, we calculated the total spectral energy (Eng), spectral entropy (Ent), and spectral kurtosis (Kurt) (Equations. (4), (5), (6)).

(4) $$E_{\mathrm{total}}=\sum P\left(u,v\right)$$

(5) $$H=-\sum_{u,v}p\left(u,v\right)\log_{2}\left(p\left(u,v\right)\right)$$

(6) $$K=\frac{\frac{1}{N_{\theta }N_{y}}\sum_{u=1}^{N_{\theta }}\sum_{v=1}^{N_{y}}{\left(P\left(u,v\right)-\mu_{P}\right)}^{4}}{{\left(\sigma_{P}^{2}\right)}^{2}}$$

where the normalized power spectrum is defined as $p\;\left(u,v\right)=\frac{P(u,v)}{E_{total}}$, and $\mu_{P}$, $\sigma_{P}^{2}$ denote the mean and variance of the 2D power spectrum, respectively.

1. Global Energy Ratios

To evaluate the distribution of spectral energy, we defined specific geometric regions within the 2D spectrum. A rectangular low‐frequency energy ratio (Rect‐LF, Equation 7) was defined to quantify energy concentration in the central low‐frequency region, indicating smoother MLC motion. Conversely, a radial high‐frequency energy ratio (Rad‐HF, Equation 8) was introduced to capture peripheral high‐frequency components corresponding to abrupt, complex modulations.

(7) $$R_{\mathrm{rect},\mathrm{LF}}=\frac{\sum_{\left(u,v\right)\in \Omega_{\mathrm{rect}}}P\left(u,v\right)}{E_{\mathrm{total}}}$$

where $\Omega_{rect}=\left\{\left(u,v\right)||u-u_{0}|\leq \lfloor \frac{N_{\theta }}{8}\rfloor ,\left|v-v_{0}\right|\leq \lfloor \frac{N_{y}}{8}\rfloor \right\}$, and $\Omega_{rect}$ defines a rectangular region in the 2D power spectrum. Here, $\left(u_{0},v_{0}\right)$ denotes the center of the spectrum, and $\left\lfloor \frac{N_{\theta }}{8}\right\rfloor$ and $\left\lfloor \frac{N_{y}}{8}\right\rfloor$ represent one‐eighth of the size along the angular axis and the spatial axis, rounded down to the nearest integer.

(8) $$R_{\mathrm{rad},\mathrm{HF}}=\frac{\sum_{\left(u,v\right)\in \Omega_{\mathrm{rad}}}P\left(u,v\right)}{E_{\mathrm{total}}}$$

where $\Omega_{\mathrm{rad},\;\mathrm{HF}}=\left\{\left(u,v\right)|r\left(u,v\right)>\alpha \;r_{max}\right\}$ defines a radial high‐frequency region, and r(u,v) denotes the Euclidean distance from frequency coordinate (u,v) to the 2D power spectrum center, and $r_{\max}$ represents the maximum radius. In this study, α was set to 0.1.

1. Note on Nomenclature

These features were extracted independently for the left leaf bank, right leaf bank, and leaf gap, denoted by the prefixes L‐, R‐, and G‐ (e.g., L‐Eng, G‐Rect‐LF).

1. Directional Spectral Features With Relative and Absolute Frequency Thresholds

To characterize the directional distribution of spectral energy, the 2D power spectra were projected onto the angular and spatial axes to obtain two 1D spectral projections (P(u) and P(v)). In this study, spectral features derived from P(u) were referred to as angular‐axis spectral features, whereas those derived from P(v) were referred to as spatial‐axis spectral features. For each axis, two types of directional features were calculated: energy ratios based on relative proportional thresholds and energy ratios based on absolute physical frequency thresholds. First, we calculated low‐ and high‐frequency energy ratios along the spatial and angular axes using predefined relative proportional thresholds (α, β) (Equations (9), (10), (11), (12)). These relative‐threshold directional features were denoted as Spa‐LF and Spa‐HF for the spatial axis, and Ang‐LF and Ang‐HF for the angular axis.

(9) $$R_{y,\mathrm{LF}}=\frac{\sum_{v\in \Omega_{\mathrm{low}}}P\left(v\right)}{E_{\mathrm{total}}}$$

(10) $$R_{y,\mathrm{HF}}=\frac{\sum_{v\in \Omega_{\mathrm{high}}}P\left(v\right)}{E_{\mathrm{total}}}$$

(11) $$R_{\theta ,\mathrm{LF}}=\frac{\sum_{u\in \Omega_{\mathrm{low}}}P\left(u\right)}{E_{\mathrm{total}}}$$

(12) $$R_{\theta ,\mathrm{HF}}=\frac{\sum_{u\in \Omega_{\mathrm{high}}}P\left(u\right)}{E_{\mathrm{total}}}$$

where the low‐frequency band is defined as $\Omega_{\mathrm{low}}=\left\{1,\text{…}\;,\alpha N\right\}$, and the high‐frequency band is defined as $\Omega_{\mathrm{high}}=\left\{\beta N,\text{…}\;,N\right\}$, with α and β being predefined proportional coefficients. In this study, α=0.2, β=0.5 were used in the spatial axis, and α=0.01 and β=0.05 were used in the angular axis. In this feasibility study, the relative thresholds α and β were empirically predefined based on qualitative inspection of the directional spectral projection profiles. These thresholds were used to characterize relatively smooth low‐frequency modulation components and rapidly varying high‐frequency modulation components, respectively.

Second, absolute physical frequency thresholds (f_th_∈{0.003,0.005,0.01,0.05,0.1}) were applied to define angular‐axis and spatial‐axis high‐frequency energy ratios based on absolute physical frequency thresholds at multiple physical‐frequency scales (Equations (13), (14), (15), (16)).

(13) $$f_{\theta }\left(u\right)=\left|\frac{u}{N_{\theta }\Delta \theta }\right|\;\deg^{-1}$$

(14) $$f_{y}\left(v\right)=\left|\frac{v}{N_{y}\Delta y}\right|\;mm^{-1}$$

(15) $$R_{\theta }\left(f_{\mathrm{th}}\right)=\frac{\sum_{f_{\theta }\left(u\right)>f_{\mathrm{th}}}P\left(u\right)}{E_{\mathrm{total}}}$$

(16) $$R_{y}\left(f_{\mathrm{th}}\right)=\frac{\sum_{f_{y}\left(v\right)>f_{\mathrm{th}}}P\left(v\right)}{E_{\mathrm{total}}}$$

The corresponding features for the left leaf bank, right leaf bank, and leaf gap were denoted as L‐Ang‐HF($f_{\mathrm{th}}$), R‐Ang‐HF($f_{\mathrm{th}}$), G‐Ang‐HF($f_{\mathrm{th}}$), and L‐Spa‐HF($f_{\mathrm{th}}$), R‐Spa‐HF($f_{\mathrm{th}}$), G‐Spa‐HF($f_{\mathrm{th}}$), respectively.

### 2.5 MU spectral analysis and feature aggregation

The MU sequence was analyzed identically as a 1D signal sampled by gantry angle. To ensure consistency with the MLC analysis, the MU signal was resampled to the same uniform gantry‐angle grid using the same linear interpolation method as that applied to the MLC leaf‐position trajectories. After mean‐centering and applying a Hann window to minimize spectral leakage, a 1D FFT was performed. We extracted total MU spectral energy (MU‐Eng), peak frequency (MU‐PF), spectral centroid (MU‐SC), spectral flatness (MU‐SF), and high‐frequency energy ratios (MU‐HF).

Finally, all spectral features were initially computed at the arc level. To generate plan‐level features for each patient plan, arc‐level features were statistically aggregated using the mean, standard deviation, and maximum values, comprehensively representing the plan's overall level, inter‐arc variability, and extreme modulation. The resulting plan‐level features were denoted by appending the suffixes Mean, Std, and Max to the corresponding arc‐level feature names, respectively (Table 1).

**TABLE 1:** Summary of plan‐level spectral features.

Category	Object	Feature	Description	Aggregation	Number of features
Leaves Spectral (global energy ratios: rectangular)	Left leaf bank (L Right leaf bank (R) Leaf Gap (G)	Object^*^‐Rect‐LF	Rectangular low‐frequency energy ratio	Mean Std Max	3 × 1 × 3 = 9
Leaves Spectral (global energy ratios: radial)		Object^*^‐Rad‐HF	Radial high‐frequency energy ratio		3 × 1 × 3 = 9
Leaves Spectral (relative thresholds)		Object^*^‐Ang‐LF; Object^*^‐Ang‐HF; Object^*^‐Spa‐LF; Object^*^‐Spa‐HF	Directional low‐frequency and high‐frequency energy ratios with relative frequency thresholds		3 × 4 × 3 = 36
Leaves Spectral (absolute thresholds)		Object^*^‐Ang‐HF(f); Object^*^‐Spa‐HF(f)	Directional high‐frequency energy ratios with physical frequency (f) thresholds		3 × 2 × 5 × 3 = 90
Leaves Spectral (global statistical features)		Object^*^‐Eng; Object^*^‐Ent; Object^*^‐Kurt	Spectral energy, entropy and kurtosis		3 × 3 × 3 = 27
MU Spectral	MU	MU‐Eng	Total spectral energy, peak frequency, spectral centroid, and spectral flatness of the MU sequence		1 × 4 × 3 = 12
		MU‐PF			
		MU‐SC			
		MU‐SF			
MU Spectral		MU‐HF(f)	MU of high‐frequency energy ratios with physical frequency thresholds		1 × 5 × 3 = 15

* Object denotes the left leaf bank (L), right leaf bank (R) and leaf gap (G) respectively. All spectral features were extracted at the arc level separately from the four objects. The mean, standard deviation, and maximum values of each arc‐level feature were computed across all arcs belonging to the same plan. The numbers of plan‐level features reported in the last column were calculated accordingly.

### 2.6 Machine learning and statistical pipeline

Alongside the proposed spectral features, 25 conventional plan complexity metrics (e.g., MU, number of CPs; detailed in Table S2) were extracted for comparison.^21^ Random Forest (RF) models were implemented using the scikit‐learn package, whereas Extreme Gradient Boosting (XGBoost) models were implemented using the XGBoost package for both GPR regression and PSQA pass/fail classification.

For the regression task, models were trained and tested using both the 3%/2 mm and 2%/1 mm gamma criteria, with global normalization and a 10% dose threshold. For the 3%/2 mm criterion, prediction errors in the test set were evaluated in three settings: (1) all test cases, (2) test cases with GPR < 95% (low GPR subset), and (3) test cases with GPR ≥ 95% (high GPR subset). This post hoc stratification of the test set results was performed to examine whether prediction accuracy differed across GPR ranges. For the classification task, plans with GPR < 97% under the 3%/2 mm gamma criterion were labeled as PSQA failures (label = 1), whereas plans with GPR ≥ 97% were labeled as passes (label = 0). The GPR distribution was clustered near the upper bound, with a mean of 98.3% and a median of 99.2%. When the TG‐218 universal tolerance limit of 95% was applied, only 36 of 481 plans (7.5%) met the criterion for PSQA failure, resulting in a highly imbalanced dataset. Previous PSQA classification studies have used more stringent gamma criteria or GPR thresholds to increase the number of PSQA failure cases and alleviate class imbalance.^21^, ^22^ Following this rationale, a 97% GPR threshold was adopted in the present study, which also partially mitigated the class imbalance. Under this GPR threshold, 61 of 481 plans (12.7%) were labeled as PSQA failures. This provided a less severely imbalanced class distribution while remaining close to the TG‐218 tolerance limit. In addition, from a pretreatment virtual PSQA perspective, a stricter threshold can help identify potential errors, allowing additional measurement‐based QA to be performed for verification when necessary.

**FIGURE 2:**
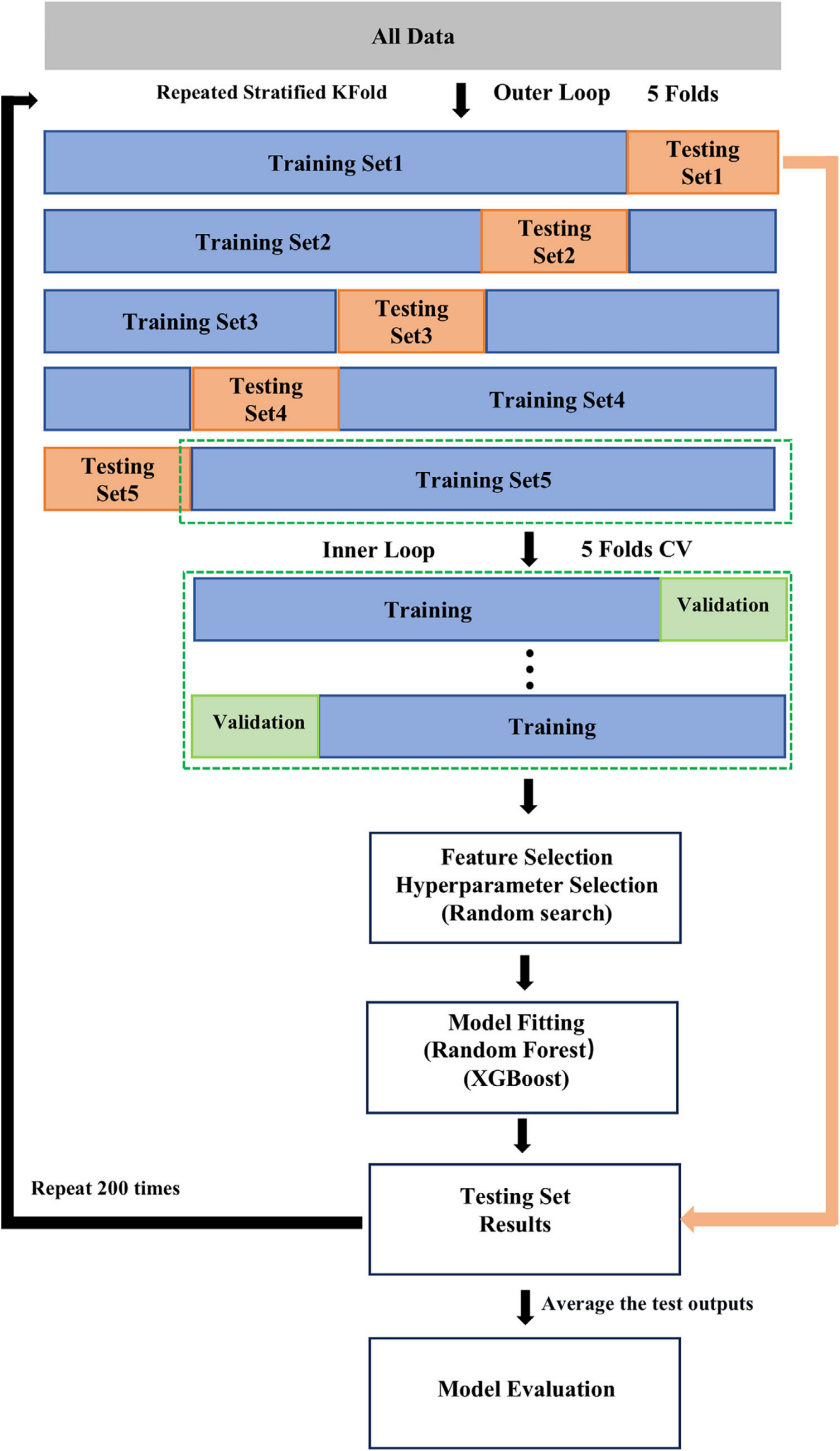
Workflow of the nested cross‐validation strategy used for model training and evaluation. The outer loop consisted of five‐fold cross‐validation repeated 200 times, while the inner loop used five‐fold cross‐validation for feature selection and hyperparameter tuning. The optimized model was then evaluated on the corresponding testing set, and the test results were averaged across all repetitions.

To reduce the risk of overfitting and data leakage, a nested cross‐validation (CV) strategy was employed (Figure 2). The outer loop utilized 200 repetitions of 5‐fold CV for robust model evaluation. Within the inner loop, we performed hyperparameter optimization via randomized search and univariate feature selection (SelectKBest, selecting 10 to 80 features). Furthermore, to address clinical class imbalance in the classification task, the Synthetic Minority Over‐sampling Technique (SMOTE) was applied strictly to the inner‐loop training sets.^23^ Feature selection, hyperparameter tuning, and SMOTE were implemented as part of a single pipeline within the inner‐loop training process, whereas the outer‐loop test folds were kept completely independent and used only for final model evaluation.

Statistical significance between extracted features and GPRs was evaluated using Spearman's rank correlation (*p* < 0.05). Highly collinear spectral features (absolute Spearman coefficient > 0.95) were filtered prior to modeling. Spearman correlation coefficients between the retained spectral features and conventional plan complexity metrics were also calculated to quantify the potential redundancy between these two feature categories. For each spectral feature group (global 2D, spatial‐axis, angular‐axis, and MU spectral), we summarized the redundancy with conventional plan complexity metrics using the median, mean, and maximum absolute Spearman coefficient, together with the proportion of feature–metric pairs reaching |r_s_| ≥ 0.7. Model performance was evaluated using MAE and RMSE for regression, and AUC, accuracy, precision, sensitivity, and specificity for classification. Finally, SHapley Additive exPlanations (Tree SHAP) was utilized to assess feature importance and provide physical interpretability to the model predictions.

We considered three feature sets to evaluate the performance of models: (1) the Complexity‐only feature set, which included plan complexity metrics only; (2) the Combined feature set, which included both plan complexity metrics and the proposed spectral features; and (3) the Spectral‐only feature set, which included spectral features only.

## 3 RESULTS

### 3.1 Spearman correlation analysis

The results of Spearman's rank correlation analysis between GPRs and all features were summarized in Table 2. The top 30 features all showed statistically significant correlations(∣r_s_∣), with the absolute correlation coefficients from 0.41 to 0.64 (p < 0.01). Notably, spectral features dominated this top‐performing subset, accounting for 19 of the 30 features, with the remainder being conventional plan complexity metrics.  Among the spectral features, the global spectral features and spatial‐axis spectral features generally showed moderate correlations, with coefficients ranging from 0.45 to 0.60 (p < 0.001). While angular‐axis spectral features showed slightly weaker correlations compared to spatial‐axis spectral features, their correlations remained statistically significant (the average *r_s_* values of G‐Ang‐HF(0.1) and G‐Ang‐HF were −0.3, −0.29, respectively).

**TABLE 2:** Mean of extracted features and their Spearman correlation coefficients with corresponding *p*‐values with respect to the GPRs. The top 30 features ranked by the Spearman correlation coefficients are shown.

Num	metric	Mean value	*r_s_*
1	EAM	6.05 × 10^−1^	−0.64
2	G‐Spa‐HF Mean	4.43 × 10^−3^	−0.61
3	Index of modulation (M)	8.77 × 10^−2^	−0.61
4	Small aperture score 20 mm (SAS 20)	4.54 × 10^−1^	−0.61
5	Average Leaf Gap	2.58 × 10^1^	0.60
6	Ratio of average area of an aperture over the area defined by jaws (AAJA)	6.50 × 10^−2^	0.60
7	G‐Eng Mean	3.04 × 10^8^	0.60
8	Average beam area (BA)	3.79 × 10^1^	0.59
9	Union area of all apertures of beam (UAA)	9.96 × 10^5^	0.57
10	G‐Spa‐HF(0.01) Mean	1.73 × 10^−1^	−0.56
11	G‐Spa‐HF(0.005) Mean	3.08 × 10^−1^	−0.55
12	L‐Rect‐LF Max	9.91 × 10^−1^	0.55
13	R‐Eng Max	8.26 × 10^8^	0.53
14	L‐Spa‐HF(0.05) Mean	3.08 × 10^−2^	−0.53
15	Small aperture score 10 mm (SAS 10)	1.54 × 10^−1^	−0.52
16	G‐Rad‐HF Mean	1.61 × 10^−3^	−0.51
17	LSV	6.70 × 10^−1^	0.51
18	R‐Rect‐LF Mean	9.91 × 10^−1^	0.51
19	L‐Eng Std	1.33 × 108	0.49
20	G‐Kurt	4.45 × 105	0.48
21	R‐Spa‐HF(0.05) Max	4.02 × 10^−2^	−0.47
22	L‐Spa‐HF(0.01) Mean	1.30 × 10^−1^	−0.46
23	R‐Eng Std	1.33 × 108	0.45
24	Average leaf travelling distance (LT)	8.66 × 102	0.45
25	MU‐Eng	2.56 × 101	−0.43
26	G‐Ent	6.66 × 10^0^	−0.43
27	Small aperture score 5 mm (SAS 5)	4.50 × 10^−2^	−0.42
28	L‐Rad‐HF Mean	1.26 × 10^−3^	−0.41
29	R‐Kurt Mean	2.65 × 105	0.41
30	L‐Kurt Mean	2.65 × 105	0.41

The correlations between spectral features and conventional plan complexity metrics were also evaluated. The median absolute spearman correlation coefficients were 0.28, 0.22, 0.19, and 0.13 for global 2D spectral features, spatial‐axis spectral features, angular‐axis spectral features, and MU spectral features, respectively. For spatial‐axis spectral features, the strongest correlation was observed between G‐Spa‐HF‐Mean and the index of modulation ($|r_{s}|$ = 0.880), and 2.49% of spatial‐axis feature–metric pairs showed strong correlations ($|r_{s}|$≥ 0.7). For angular‐axis spectral features, the maximum correlation coefficient was 0.656, and no angular‐axis feature–metric pair reached strong correlations.

### 3.2 Prediction evaluation and feature importance

**FIGURE 3:**
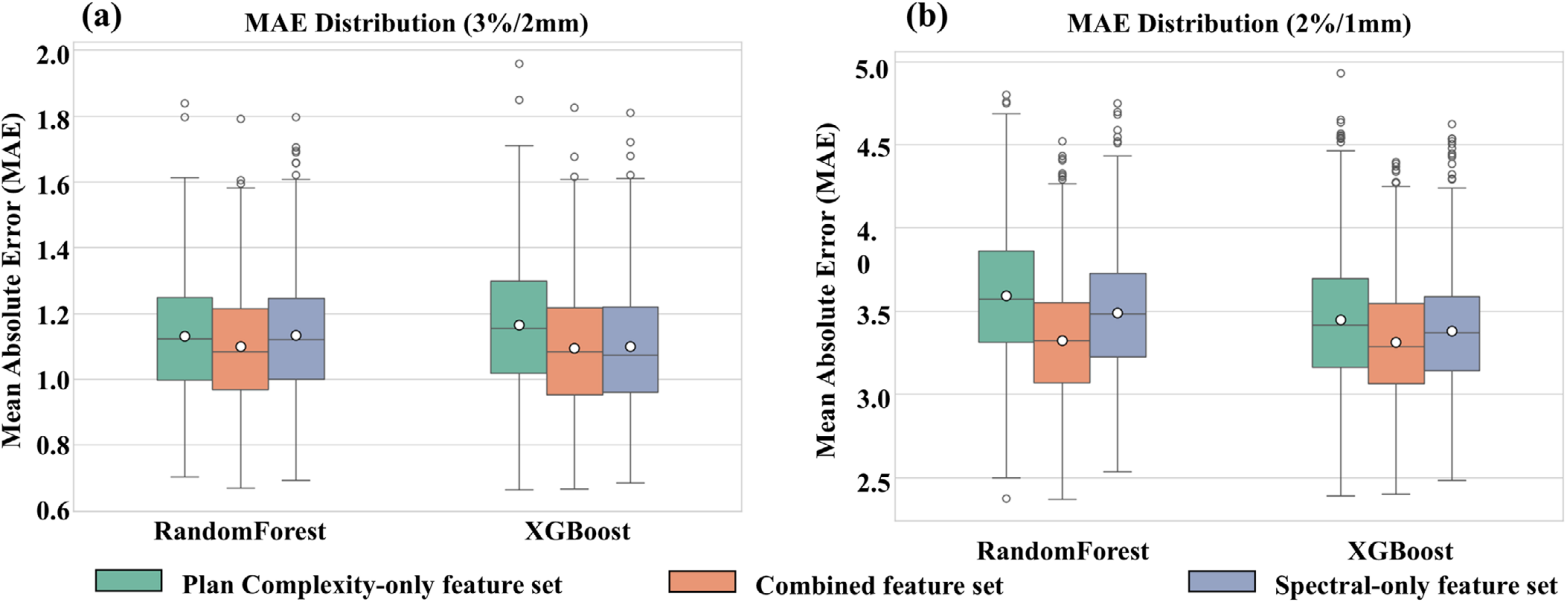
Boxplots of mean absolute error (MAE) for the Random Forest and XGBoost regression models using three feature input sets: plan complexity‐only, combined, and spectral‐only. Results are shown under the (a) 3%/2 mm and (b) 2%/1 mm gamma criteria.

Across all evaluations, the combined feature set generally yielded the lowest MAE for both RF and XGBoost models, although the improvement over the individual feature sets was modest. (Table 3, Figure 3). Under the standard 3%/2 mm GPR criterion, the combined XGBoost model achieved the lowest MAE of 1.095 ± 0.182. When evaluated under the more stringent 2%/1 mm criterion, MAE values naturally increased (3.312 ± 0.345 for XGBoost) but maintained the same inter‐model performance hierarchy.

**TABLE 3:** Prediction performance (MAE and RMSE) of RF and XGBoost models using different feature sets under different gamma evaluation standards. Results are shown as mean ± standard deviation over 200 repetitions.

			Features type		
ML model	Gamma criteria	Performance	Plan complexity‐only feature set	Combined feature set	Spectral‐only feature set
Random Forest	3%/2mm (200 repetitions)	MAE(%)	1.130 ± 0.178	1.099 ± 0.175	1.133 ± 0.179
		RMSE(%)	2.241 ± 0.643	2.134 ± 0.646	2.193 ± 0.648
XGBoost		MAE(%)	1.166 ± 0.195	1.095 ± 0.182	1.100 ± 0.189
		RMSE(%)	2.336 ± 0.655	2.169 ± 0.657	2.174 ± 0.700
Random Forest	2%/1mm (200 repetitions)	MAE(%)	3.592 ± 0.407	3.324 ± 0.358	3.488 ± 0.368
		RMSE(%)	5.542 ± 0.897	5.132 ± 0.839	5.282 ± 0.840
XGBoost		MAE(%)	3.446 ± 0.392	3.312 ± 0.345	3.379 ± 0.348
		RMSE(%)	5.370 ± 0.909	5.093 ± 0.786	5.159 ± 0.789
Random Forest	3%/2mm	MAE(%)	4.986 ± 1.817	4.613 ± 1.870	4.729 ± 1.917
	Low GPRs test sets	RMSE(%)	6.598 ± 2.683	6.215 ± 2.751	6.375 ± 2.771
XGBoost	(GPRs < 95% with 200 repetitions)	MAE(%)	5.212 ± 1.810	4.835 ± 1.861	5.113 ± 1.877
		RMSE(%)	6.732 ± 2.738	6.383 ± 2.724	6.618 ± 2.760
Random Forest	3%/2mm	MAE(%)	0.813 ± 0.111	0.808 ± 0.102	0.835 ± 0.107
		RMSE(%)	1.266 ± 0.240	1.221 ± 0.179	1.260 ± 0.197
XGBoost	High GPRs test sets (GPRs > 95% with 200 repetitions)	MAE(%)	0.833 ± 0.127	0.786 ± 0.111	0.769 ± 0.101
		RMSE(%)	1.371 ± 0.307	1.210 ± 0.247	1.140 ± 0.228

**FIGURE 4:**
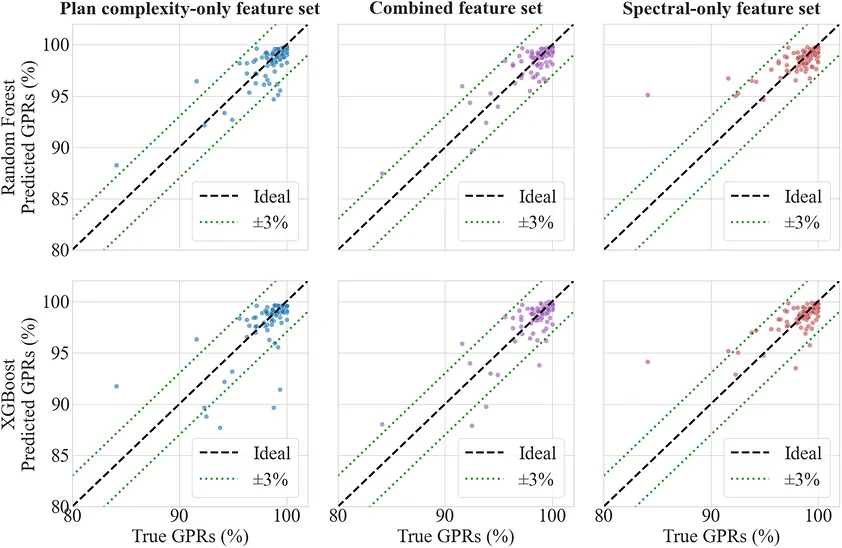
Scatter plots of predicted versus true GPRs for the Random Forest (RF, top row) and XGBoost (bottom row) models using three different feature sets: plan complexity metrics (left column), combined spectral and plan complexity features (middle column), and spectral features (right column). The dashed black lines indicate the identity line, and the dotted green lines represent ± 3% deviation bands. Each point corresponds to one test case from a representative test fold.

Importantly, models utilizing the spectral‐only feature set performed comparably to those trained strictly on conventional plan complexity‐only set (e.g., XGBoost MAE under 3%/2 mm criterion: 1.100 vs. 1.166). In the clinically challenging low‐GPR subset (< 95%), the combined feature set achieved the lowest MAE, with a value of 4.613 ± 1.870 (RF models). Figure 4 illustrates the overall agreement between predicted and measured GPRs.

### 3.3 Classification performance evaluation

**FIGURE 5:**
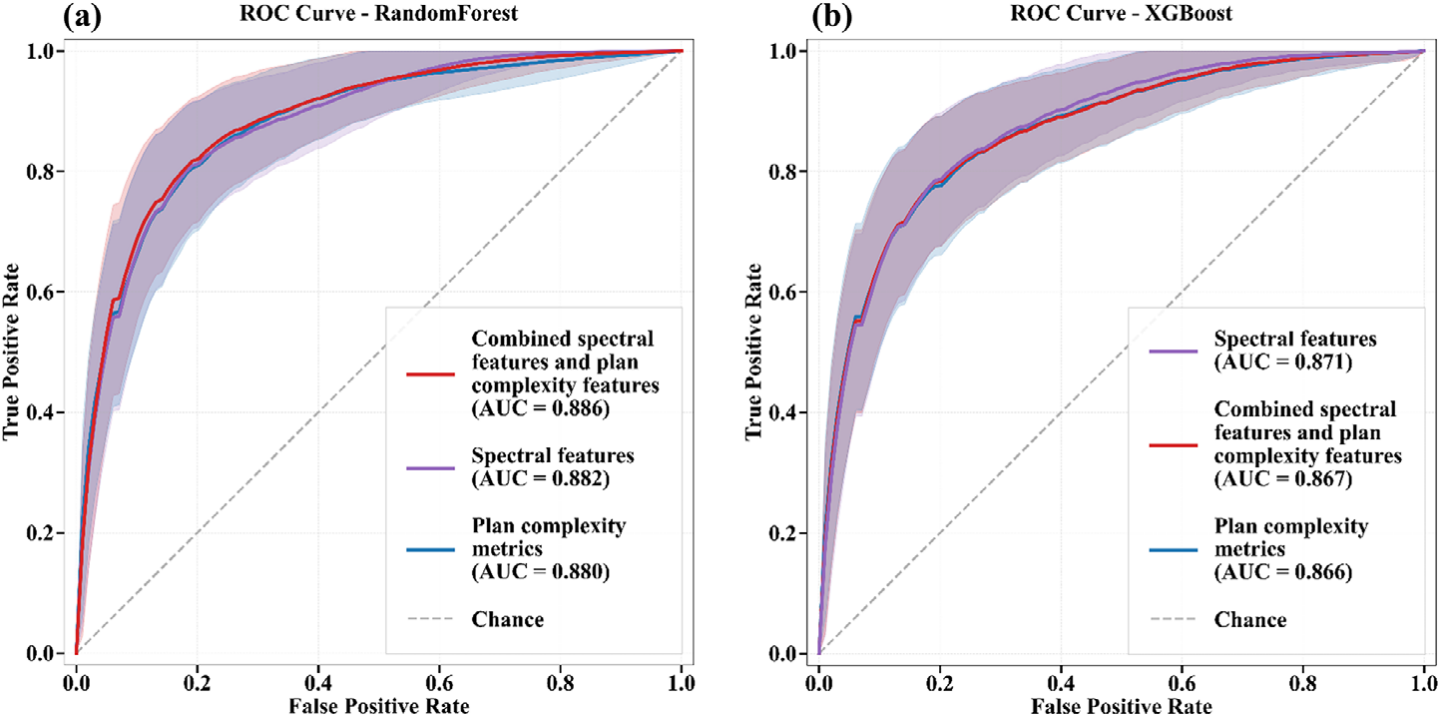
Receiver operating characteristic (ROC) curves of the classification models using three feature input sets: plan complexity metrics, spectral features, and the combined feature set. PSQA failure was defined as GPR < 97% under the 3%/2 mm gamma criterion. Results are shown for (a) the Random Forest classifier and (b) the XGBoost classifier. The shaded regions indicate ± 1 standard deviation across 200 repeated five‐fold cross‐validation runs.

In the PSQA pass/fail classification task, the RF model using the combined feature set yielded an AUC of 0.886 ± 0.043, an accuracy of 0.844 ± 0.066, and an F1 score of 0.602 ± 0.094 (Table 4, Figure 5). The corresponding AUC values for the complexity‐only and spectral‐only feature sets were 0.880 ± 0.046 and 0.882 ± 0.042, respectively. Paired Wilcoxon signed‐rank tests showed that the AUC difference between the combined and complexity‐only feature sets was statistically significant for RF (*p* < 0.001). For XGBoost, the combined feature set yielded an AUC of 0.867 ± 0.047, compared with 0.866 ± 0.050 for the complexity‐only feature set and 0.871 ± 0.045 for the spectral‐only feature set. The AUC difference between the combined and complexity‐only feature sets was not statistically significant for XGBoost (*p* = 0.544).

**TABLE 4:** Classification performance of the Random Forest and XGBoost models using three feature sets. PSQA failure was defined as GPR < 97% under the 3%/2 mm criterion and labeled as 1, whereas pass was defined as GPR ≥97% and labeled as 0; the original dataset included 61 failure cases and 420 pass cases. Values are reported as mean ± standard deviation across 200 repetitions of five‐fold cross‐validation.

		Features type		
ML model	Performance	Plan complexity metrics	Combined spectral features and plan complexity features	Spectral features
Random Forest	AUC	0.880 ± 0.046	**0.886** ± **0.043**	0.882 ± 0.042
	Accuracy	0.832 ± 0.069	**0.844** ± **0.066**	0.839 ± 0.065
	Precision	0.458 ± 0.117	**0.480** ± **0.124**	0.468 ± 0.117
	Sensitivity	**0.864** ± **0.089**	0.861 ± 0.093	0.854 ± 0.093
	Specificity	0.828 ± 0.085	**0.841** ± **0.082**	0.837 ± 0.080
	F1	0.586 ± 0.093	**0.602** ± **0.094**	0.592 ± 0.090
XGBoost	AUC	0.866 ± 0.050	0.867 ± 0.047	0.871 ± 0.045
	Accuracy	0.834 ± 0.073	0.835 ± 0.073	0.830 ± 0.073
	Precision	0.465 ± 0.131	0.467 ± 0.129	0.457 ± 0.125
	Sensitivity	0.833 ± 0.103	0.832 ± 0.104	0.842 ± 0.101
	Specificity	0.834 ± 0.091	0.835 ± 0.091	0.828 ± 0.091
	F1	0.581 ± 0.098	0.583 ± 0.095	0.577 ± 0.093

### 3.4 SHAP analysis

**FIGURE 6:**
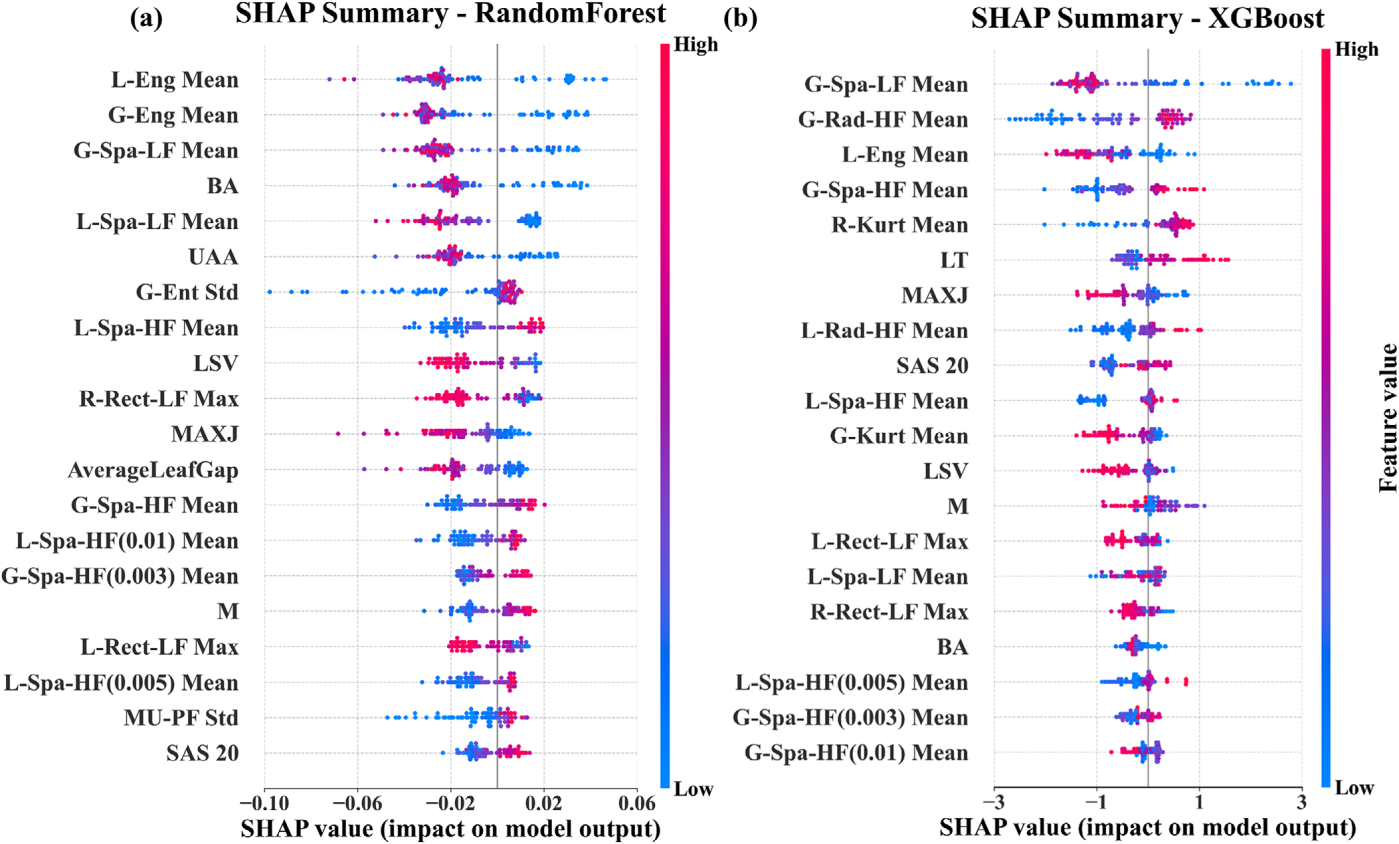
SHAP summary plots of the classification models using the combined feature set for (a) Random Forest and (b) XGBoost. Features are ranked by their overall contribution to model output. Each point represents one sample, and color indicates the feature value from low to high. Positive SHAP values indicate contributions toward failure prediction, whereas negative SHAP values indicate contributions toward pass prediction.

To elucidate model decision‐making, SHAP analyses were conducted for both classifiers (Figure 6). In both RF and XGBoost models, spectral features ranked among the most important contributors to the classification decision. Specifically, global statistical features (e.g., L‐Eng, G‐Eng) and spatial‐axis spectral features (e.g., G‐Spa‐LF, G‐Spa‐HF) emerged as the most critical predictors.

With the output labels defined as failure (1) and pass (0), positive SHAP values indicate contributions toward failure prediction, whereas negative SHAP values indicate contributions toward pass prediction. In both models, larger values of low‐frequency and energy‐related spectral features (e.g., G‐Spa‐LF, L‐Eng) were generally associated with negative SHAP values, contributing to ‘pass’ predictions. Conversely, higher values of high‐frequency spectral features (e.g., G‐Spa‐HF, G‐Rad‐HF) predominantly yielded positive SHAP values, signifying an increased risk of PSQA failure.

## 4 DISCUSSION

In this study, we proposed and evaluated a machine‐learning approach using FFT‐based frequency‐domain spectral features for predicting VMAT GPRs and classifying PSQA failures. By applying FFT‐based spectral analysis to MLC leaf position trajectories and MU signals, we extracted spectral features that characterized modulation complexity from a frequency‐domain perspective. Conventional plan complexity metrics typically quantify specific geometric, aperture‐related, or MLC motion‐related properties of VMAT plans; therefore, each metric tends to capture only a particular aspect of plan modulation complexity. For example, AAV primarily reflects variations in aperture area across CPs, LSV characterizes the regularity of MLC leaf arrangements and small aperture score (SAS) quantifies the proportion of small field openings. In contrast, the proposed FFT‐based spectral analysis framework represents MLC leaf trajectories and MU sequences as angular‐domain signals and incorporates them into a unified frequency‐domain analytical framework. From this perspective, plan modulation complexity can be characterized by analyzing spectral power distributions across multiple analytical dimensions, including global, spatial‐axis, angular‐axis, and MU‐related components. For each analytical dimension, spectral descriptors such as spectral energy, spectral entropy, and low‐ and high‐frequency energy ratios were extracted to quantify the corresponding frequency‐domain modulation characteristics. As a feasibility validation, the spectral features were significantly associated with measured GPRs and achieved standalone predictive performance broadly comparable to that of established plan complexity metrics, whereas their combination with conventional metrics yielded modest improvements. These findings suggest that FFT‐based spectral feature analysis provides a useful frequency‐domain perspective for quantifying VMAT plan complexity and may support machine‐learning‐based prediction of PSQA outcomes.

For the classification task, combining FFT‐based spectral features to conventional complexity metrics resulted in a statistically significant AUC improvement for the RF classifier, although the absolute difference was small. In contrast, no significant AUC improvement was observed for XGBoost. These findings indicate that the incremental benefit of the combined feature set was limited and model‐dependent, and should be interpreted cautiously. Nevertheless, the results support FFT‐based spectral analysis as a frequency‐domain framework for characterizing VMAT plan modulation complexity, which may complement conventional complexity metrics in virtual PSQA modeling.

The 2D global spectral features included spectral energy, spectral entropy, and low‐ and high‐frequency energy ratios. According to Parseval's theorem, the signal energy in the angular–spatial domain is equivalent to that in the 2D frequency domain. The larger spectral energy may reflect larger signal amplitude or geometric extent and may have different physical meanings depending on whether it is derived from leaf bank position, leaf gap, or MU modulation. For the MLC energy features G‐Eng and R‐Eng, this geometric extent may be related to aperture‐scale characteristics such as mean leaf gap^24^ and average beam area.^25^ Larger leaf‐bank apertures and beam areas are associated with wider MLC openings and smoother dose gradients, which reduce the dosimetric uncertainties associated with small‐field effects and MLC positioning, thereby improving delivery accuracy. This may partly explain the positive correlations of G‐Eng and R‐Eng with GPR. In contrast, MU‐Eng was derived from the angular MU signal, which represents the MU delivered at each gantry angle. Because the signal was mean‐subtracted before FFT analysis, MU spectral energy is closely related to the variance of the angular MU values. Greater angular MU spectral energy indicates more nonuniform modulation and is generally associated with greater delivery uncertainty, which may partly explain its negative correlation with GPR. In addition, low‐frequency components likely reflect slowly varying and lower modulation complexity, whereas high‐frequency components indicate higher modulation complexity. This interpretation is consistent with our correlation results, in which low‐frequency energy‐ratio features such as L‐Rect‐LF and R‐Rect‐LF were positively correlated with GPRs, whereas high‐frequency energy‐ratio features such as G‐Rad‐HF were negatively correlated with GPRs.

Beyond global features, spatial‐axis spectral features provided highly sensitive indicators of inter‐leaf variability. These features characterize inter‐leaf variations between adjacent MLC leaves during delivery. Our redundancy analysis showed limited overlap between the spatial‐axis features and conventional plan complexity metrics. Among the directional features, G‐Spa‐HF Mean showed the strongest association with GPR ($r_{s}=-0.61$) among all spectral features, outperformed only by the EAM.^26^ This prominence likely stems from the fact that spatial‐axis high‐frequency components effectively capture rapid fluctuations in the leaf gap between adjacent leaves, serving as a sensitive indicator of aperture irregularity. Similar findings were also observed for the corresponding spatial‐axis high‐frequency features of the left and right leaf banks. This further suggests that a larger proportion of high‐frequency energy along the spatial axis reflects increased variability in aperture shape and leaf positions. Conversely, when the aperture approaches a near‐rectangular shape, the spectral content is concentrated mainly in the near‐zero and low‐frequency components.

Interestingly, angular‐axis spectral features generally exhibited weaker correlations with GPR than the corresponding spatial‐axis spectral features. This discrepancy may reflect the influence of the linear accelerator's dynamic delivery control mechanisms. When more intensive leaf motion is required, the gantry speed can be reduced to keep leaf speed and acceleration within allowable limits, which may partly weaken the association between angular‐axis spectral features and GPR.^27^ Because the MLC trajectories and MU signals were analyzed on a uniform gantry‐angle grid rather than a uniform time grid, the angular‐axis spectral features quantify modulation per degree of gantry rotation and do not incorporate the delivery time associated with each angular interval. Consequently, when the gantry slows down, the leaf motion within a given angular interval may be spread over a longer delivery time, shifting it toward lower temporal frequencies. This may weaken the association between angular‐axis spectral features and GPR. While this interpretation remains to be verified with delivery log‐file data, the high‐frequency angular‐axis features nonetheless remained significantly negatively correlated with GPR despite their relatively weak correlations. Park et al. reported a significant negative correlation between the MLC acceleration modulation index ($MI_{a}$) and GPR. ^17^ In our study, a similar relationship was observed for angular‐axis high‐frequency features, such as G‐Ang‐HF(0.1) ($r_{s}=-0.31$) and G‐Ang‐HF ($r_{s}=-0.29$), which were also negatively correlated with GPR. Together, these findings support the interpretation that more abrupt and highly dynamic modulation patterns may be associated with reduced delivery accuracy.

The interpretability and potential relevance of these spectral features were further supported by SHAP analysis. Higher values of high‐frequency features tended to shift model predictions toward QA failure, consistent with the interpretation that increased high‐frequency modulation, reflecting more complex MLC motion and greater aperture irregularity, may be associated with poorer deliverability. In contrast, low‐frequency and energy‐related features tended to favor pass prediction, which is consistent with more stable and regular aperture patterns. These nonlinear feature attributions were also in agreement with the Spearman correlation analysis. Together, these findings suggest that spectral features capture physically meaningful characteristics of VMAT modulation relevant to plan deliverability.

Several recent studies have used CP‐level MLC and MU information as inputs to train deep learning models for predicting the deliverability of VMAT plans. For example, Cho et al. generated three‐channel 3D images from DICOM RT Plan files by incorporating the beam field open size together with MLC and MU variations across CPs, and trained a DenseNet‐based classifier to predict VMAT plan deliverability. This study demonstrated that deep learning networks can model nonlinear relationships in CP‐level MLC leaf and MU sequential features, thereby effectively characterizing the modulation complexity of VMAT plans. The FFT‐based approach used in the present study also analyzes MLC leaf trajectories and MU signals stored in CPs, but from a frequency‐domain perspective. Unlike end‐to‐end deep learning models that learn latent feature representations implicitly from CP‐level inputs, the proposed method extracts handcrafted spectral features in the frequency domain. By defining a series of spectral features in the frequency domain, the proposed method explicitly quantifies the frequency characteristics of MLC leaf motion and MU modulation, thereby providing a frequency‐domain characterization of VMAT plan complexity. Such spectral information is not explicitly captured by conventional plan complexity metrics, which typically summarize plan modulation into single‐value descriptors, and may remain less transparent when implicitly learned by end‐to‐end deep learning models.

Compared with deep learning approaches, the primary strength of the proposed FFT‐based machine‐learning framework may not lie in superior predictive performance, but rather in its improved interpretability, lower computational cost, and suitability for relatively small clinical datasets. Because the proposed spectral features are explicitly defined, they can be directly compared with conventional plan complexity metrics and further interpreted using SHAP analysis. This allows model predictions to be linked to physically relevant modulation characteristics, such as the frequency‐domain characteristics of MLC leaf motion and MU. In this sense, FFT‐based spectral features and deep‐learning‐based representations should be regarded as complementary rather than competing approaches. In addition, frequency‐domain representations have already been incorporated into deep learning‐based medical image segmentation and have demonstrated promising segmentation performance. Therefore, future work could explore incorporating two‐dimensional FFT spectra into deep learning models to provide frequency‐domain guidance, allowing neural networks to learn simultaneously from CP‐level modulation patterns and their corresponding spectral representations.

Several limitations of this study should be acknowledged. First, all data were acquired from a single linac, and external validation was not performed. In addition, thoracic cases accounted for 57.4% of the dataset, resulting in an imbalanced distribution of treatment sites. Therefore, the robustness and generalizability of the proposed model across different treatment sites remain to be further validated in larger multicenter datasets with more balanced treatment site distributions. Second, the proposed spectral features may be affected by TPS‐specific CP spacing and plan parameterization. Although MLC leaf trajectories and MU signals were resampled onto a uniform gantry angle grid before FFT analysis, differences in the original CP distribution and interpolation characteristics across TPSs may still influence the spectral features. Therefore, larger multicenter external validation studies involving multiple TPSs are needed before the generalizability and practical clinical relevance of the proposed spectral features can be established. Third, the low‐frequency and high‐frequency energy‐ratio thresholds, as well as the physically defined frequency thresholds used in the spectral analysis, were empirically predefined in this feasibility study based on the qualitative characteristics of the directional spectral projection profiles. Sensitivity analyses were not performed to evaluate the robustness of model performance or the associations between spectral features and GPR across different threshold settings. Further work is needed to determine whether these settings can be optimized through systematic sensitivity analyses or data‐driven approaches. Another limitation is that the proposed spectral features were not directly validated against realized delivery‐error metrics derived from delivery log files or delivery reconstruction data. Therefore, although this study demonstrates associations between spectral features and PSQA outcomes, their physical interpretation should primarily be understood in terms of plan‐level modulation characteristics related to PSQA performance, rather than as direct evidence of specific delivery‐error mechanisms, such as MLC positional errors, leaf‐speed variations, or gantry‐speed variations. Future studies incorporating delivery log‐file analysis or delivery reconstruction are needed to provide stronger physical validation of the proposed frequency‐domain features. In addition, all treatment plans were recalculated on the ArcCHECK phantom using a 3 mm dose calculation grid. According to the SNC Patient software documentation, imported TPS dose data are interpolated to a 1 mm grid for DTA and gamma analysis, and interpolation in high dose gradient regions is more accurate when the initial grid spacing of the imported dose data is ≤3 mm. However, interpolation cannot fully recover spatial information that is absent from the original dose calculation grid. Therefore, the 3 mm dose calculation grid may still limit the spatial resolution of the calculated dose distribution and may reduce the sensitivity of PSQA evaluation to small delivery errors, particularly for highly complex plans evaluated using the 3%/2 mm and 2%/1 mm gamma criteria.

## 5 CONCLUSION

FFT‐based frequency‐domain spectral analysis provides a physically interpretable framework for quantifying VMAT modulation complexity from a frequency‐domain perspective. These features showed feasible performance for GPR prediction and PSQA failure classification. These findings support their potential utility as interpretable inputs for virtual VMAT PSQA. Future work will further validate their robustness and generalizability in multicenter datasets, with the aim of developing a more comprehensive and reliable framework for virtual PSQA assessment.

## AUTHOR CONTRIBUTION

Bing Yan conceived the experiments. Bing Yan and Chichao Zheng acquired and analyzed the data for the work. Bing Yan, Hu Peng and Aidong Wu designed the study and analyzed the result. Bing Yan, Xudong Xue and Aidong Wu participated in writing manuscript. The final version of the manuscript has been reviewed and approved for publication by all authors.

## CONFLICT OF INTEREST STATEMENT

The authors declare no conflicts of interest.

## ETHICS STATEMENT

This study was conducted in accordance with the Declaration of Helsinki and was approved by the Ethics Committee of the First Affiliated Hospital of USTC (Approval No. 2022‐RE‐177). The requirement for informed consent was waived due to the retrospective nature of the study.

## Supporting information




**Supporting Information**: acm270772‐sup‐0001‐Table S1.docx

**Supporting Information**: acm270772‐sup‐0002‐Table S2.docx

## Data Availability

The data that support the findings of this study are available from the corresponding author upon reasonable request.
