# Supplementary material for: Fast fourier transform based spectral features for machine learning prediction of gamma passing rates in virtual VMAT PSQA: A feasibility study
Source: J Appl Clin Med Phys. 2026 Aug 27;27(9):e70772. doi: 10.1002/acm2.70772 (PMC13522387; doi:10.1002/acm2.70772)
Supplement: Supplementary file 1 [file ACM2-27-e70772-s001.docx]

**Supplementary Table S1. Dataset characteristics and GPR distributions**

| Characteristic | Value |
| --- | --- |
| Total number of plans | 481 |
| **Treatment planning system, n (%)** |  |
| Monaco | 437 (90.9%) |
| Pinnacle | 44 (9.1%) |
| **Anatomical site, n (%)** |  |
| Brain | 40 (8.3%) |
| Head and neck | 43 (8.9%) |
| Thorax | 276 (57.4%) |
| Abdomen | 87 (18.1%) |
| Pelvis | 32 (6.7%) |
| Other | 3 (0.6%) |
| **Number of arcs per plan** |  |
| Mean ± SD | 2.19 ± 0.55 |
| Median | 2 |
| Range | 2–4 |
| **Number of arcs distribution, n (%)** |  |
| 2 arcs | 424 (88.1%) |
| 3 arcs | 21 (4.4%) |
| 4 arcs | 36 (7.5%) |
| **Number of control points per plan** |  |
| Mean ± SD | 276.5 ± 79.8 |
| Median [IQR] | 289 [214–335] |
| Range | 66–698 |
| **Classification task labels (3%/2 mm), n (%)** |  |
| Failure, GPR <97% | 61 (12.7%) |
| Pass, GPR ≥97% | 420 (87.3%) |
| **3%/2 mm global normalization GPR, %** |  |
| Mean ± SD | 98.33 ± 2.85 |
| Median [IQR] | 99.2 [98.2–99.7] |
| Range | 69.2–100.0 |
| GPR <90%, n (%) | 10 (2.1%) |
| 90% ≤ GPR <95%, n (%) | 26 (5.4%) |
| GPR ≥95%, n (%) | 445 (92.5%) |
| Low-GPR, GPR <95%, n (%) | 36 (7.5%) |
| High-GPR, GPR ≥95%, n (%) | 445 (92.5%) |
| **2%/1 mm global normalization GPR, %** |  |
| Mean ± SD | 90.65 ± 8.36 |
| Median [IQR] | 93.1 [88.3–96.0] |
| Range | 42.5–99.7 |
| GPR <90%, n (%) | 153 (31.8%) |
| 90% ≤ GPR <95%, n (%) | 164 (34.1%) |
| GPR ≥95%, n (%) | 164 (34.1%) |
