# Supplementary material for: Fast fourier transform based spectral features for machine learning prediction of gamma passing rates in virtual VMAT PSQA: A feasibility study
Source: J Appl Clin Med Phys. 2026 Aug 27;27(9):e70772. doi: 10.1002/acm2.70772 (PMC13522387; doi:10.1002/acm2.70772)
Supplement: Supplementary file 2 [file ACM2-27-e70772-s002.docx]

Supplementary Table S2. Conventional plan complexity metrics extracted for model comparison.

| Num | Complexity metric |
| --- | --- |
| 1 | Total MU per beam |
| 2 | Plan normalized MU (PMU) |
| 3 | Number of segments |
| 4 | Average leaf gap |
| 5 | Ratio of average area of an aperture over the area defined by jaws (AAJA) |
| 6 | Maximum of x-y jaw positions (MAXJ) |
| 7 | Percentage of jaw tracking |
| 8 | Index of modulation (M) |
| 9 | Leaf sequence variability (LSV) |
| 10 | Aperture area variability (AAV) |
| 11 | Modulation complexity score (MCS) |
| 12 | VMAT modulation complexity score (MCSV) |
| 13 | Average leaf travelling distance (LT) |
| 14 | Combination of LT and MCS (LTMCS) |
| 15 | Average beam area (BA) |
| 16 | Average beam irregularity (BI) |
| 17 | Average beam modulation (BM) |
| 18 | Edge metric (EM) |
| 19 | Edge area metric (EAM) |
| 20 | Mean asymmetry distance (MAD) |
| 21 | Union area of all apertures of beam (UAA) |
| 22 | Small aperture score 2 mm (SAS 2 mm) |
| 23 | Small aperture score 5 mm (SAS 5 mm) |
| 24 | Small aperture score 10 mm (SAS 10 mm) |
| 25 | Small aperture score 20 mm (SAS 20 mm) |

Twenty-five conventional plan complexity metrics were extracted from each VMAT plan and used as baseline features for comparison with the proposed FFT-based spectral features.
